# MARCKS protein overexpression in inflammatory breast cancer

**DOI:** 10.18632/oncotarget.14057

**Published:** 2016-12-21

**Authors:** Maroua Manai, Jeanne Thomassin-Piana, Amor Gamoudi, Pascal Finetti, Marc Lopez, Radhia Eghozzi, Sinda Ayadi, Olfa Ben Lamine, Mohamed Manai, Khaled Rahal, Emmanuelle Charafe-Jauffret, Jocelyne Jacquemier, Patrice Viens, Daniel Birnbaum, Hamouda Boussen, Max Chaffanet, François Bertucci

**Affiliations:** ^1^ Département d’Oncologie Moléculaire, Centre de Recherche en Cancérologie de Marseille, Aix Marseille Université, Marseille, France; ^2^ Département de Bio-Pathologie, Institut Paoli-Calmettes, Marseille, France; ^3^ UFR de Médecine, Aix Marseille Université, Marseille, France; ^4^ Département d’Oncologie Médicale, Institut Paoli-Calmettes, Marseille, France; ^5^ Département de Biologie, Unité de Biochimie et Biologie Moléculaire, Faculté des Sciences de Tunis, Université de Tunis El Manar, Tunisie; ^6^ Département d’Oncologie Médicale, Institut Salah Azaiez, Tunis, Tunisie; ^7^ Service d’Oncologie Médicale, Hôpital l’Ariana, Tunis, Tunisie

**Keywords:** expression, immunohistochemistry, inflammatory breast cancer, MARCKS, survival

## Abstract

**Background:**

Inflammatory breast cancer (IBC) is the most aggressive form of locally-advanced breast cancer. Identification of new therapeutic targets is crucial. We previously reported *MARCKS* mRNA overexpression in IBC in the largest transcriptomics study reported to date. Here, we compared MARCKS protein expression in IBC and non-IBC samples, and searched for correlations between protein expression and clinicopathological features.

**Results:**

Tumor samples showed heterogeneity with respect to MARCKS staining: 18% were scored as MARCKS-positive (stained cells ≥ 1%) and 82% as MARCKS-negative. MARCKS expression was more frequent in IBC (36%) than in non-IBC (11%; *p* = 1.4E−09), independently from molecular subtypes and other clinicopathological variables. We found a positive correlation between protein and mRNA expression in the 148/502 samples previously analyzed for MARCKS mRNA expression. MARCKS protein expression was associated with other poor-prognosis features in the whole series of samples such as clinical axillary lymph node or metastatic extension, high pathological grade, ER-negativity, PR-negativity, HER2-positivity, and triple-negative and HER2+ statutes. In IBC, MARCKS expression was the sole tested variable associated with poor MFS.

**Materials and Methods:**

We retrospectively analyzed MARCKS protein expression by immunohistochemistry in 502 tumors, including 133 IBC and 369 non-IBC, from Tunisian and French patients. All samples were pre-therapeutic clinical samples. We searched for correlations between MARCKS expression and clinicopathological features including the IBC versus non-IBC phenotype and metastasis-free survival (MFS).

**Conclusions:**

MARCKS overexpression might in part explain the poor prognosis of IBC. As an oncogene associated with poor MFS, MARCKS might represent a new potential therapeutic target in IBC.

## INTRODUCTION

Inflammatory breast cancer (IBC) is the most aggressive form of locally-advanced breast cancer. Classified T4d in the AJCC (American Joint Committee on Cancer) staging system, IBC is clinically defined by signs of inflammation (erythema, edema, warming, “peau d’orange”) arising quickly and involving more than one-third of the breast [1]. IBC is associated with a high metastatic propensity, and despite the introduction of multimodality treatment, the 5-year survival remains around 50%, inferior to that observed in non-IBC. IBC differs from non-IBC at many other levels. At the epidemiological level, IBC is more frequent in geographic areas such as North Africa [1, 2]. At the pathological level, IBCs are more frequently than non-IBC associated with ductal type, axillary lymph node invasion, high grade, ER and PR-negativity and HER2-positivity. The presence of tumor emboli in dermal lymphatic vessels is the pathological hallmark of IBC, but is neither mandatory nor sufficient for diagnosis. At the molecular level, higher incidence of certain alterations has been reported in IBC, such as EGFR overexpression, *TP53* mutations, high proliferation and angiogenesis levels, and overexpression of E-cadherin and eIFG4I (see [3] for review).

To better understand the pathophysiology of IBC, high-throughput molecular profiling was applied during the two last decades to preclinical models and clinical samples, initially focused on mRNA expression profiling [4], then on other technologies [5, 6]. These studies showed that IBC is a heterogeneous disease comprising all molecular subtypes previously described in non-IBC, but with more frequent aggressive subtypes, and that a molecular signature of IBC may be established. In 2008, we and others founded the World IBC Consortium to foster collaboration between research groups focusing on IBC. The first project was to redefine the molecular profile of IBC using an unprecedented number of samples. We gathered gene expression profiles of 389 clinical tumor samples, including 137 IBC and 252 non-IBC, which remains by far the largest series of IBC samples ever analyzed [7]. That allowed for the first time to take into account in the supervised analysis the unbalance in term of molecular subtypes between IBC and non-IBC. We identified a robust 79-gene IBC expression signature independent from the molecular subtypes. Among the top three genes overexpressed in IBC *versus* non-IBC, was *MARCKS*, which encodes for the myristoylated alanine-rich protein kinase C substrate.

MARCKS is a substrate of protein kinase C (PKC) that shuttles between the plasma membrane and cytoplasm in a PKC phosphorylation-dependent manner. It is ubiquitously expressed in various tissues [8] and is involved in cell adhesion, motility through the regulation of the actin cytoskeleton, cytokine secretion, and phagocytosis of inflammatory cells [9]. In some tissues, MARCKS phosphorylation is regulated by other kinases such as RHO kinases and MAP kinases [10, 11], the activation of which has been linked to metastasis [12]. Several recent data have shown the involvement of MARCKS in cancer aggressiveness, notably metastatic process and therapeutic resistance [13–18], and the efficiency of therapeutic inhibition of MARCKS [19, 20]. Such data, combined with MARCKS overexpression in IBC *versus* non-IBC, suggested that MARCKS might be a relevant target in IBC.

Here, as a first step and to validate our initial observation, we evaluated and compared MARCKS protein expression in 133 IBC and 369 non-IBC tumor samples collected from French and Tunisian patients. Correlations were established between protein expression and clinicopathological features.

## RESULTS

### Breast cancer population and clinicopathological features

The clinicopathological features of all samples (*N* = 502), IBC (*N* = 133) and non-IBC (*N* = 369) separately, are shown in Table 1. Patients with IBC were younger than patients with non-IBC (*p* = 6.1E-10). IBC cases were higher clinical stage with more frequent axillary lymph node involvement (*p* = 3.5E-22) and metastasis at diagnosis (*p* = 1.8E-19). IBC samples showed more frequently than non-IBC samples pejorative pathological prognostic features: ductal type (*p* = 1.2E-05), high grade (*p* < 1.0E-06), ER-negativity (*p* = 5.7E-04), PR-negativity (*p* = 1.3E-02), and HER2-positivity (*p* = 2.1E-17). Finally, the 5-year MFS was 53% (95% CI, 43–66) in patients with IBC and 81% (95% CI, 77–85) in patients with non-IBC (*p* = 1.3E-11). Altogether, these observations confirmed the clinical coherence of our series.

**Table 1:** Clinicopathological characteristics of breast cancer samples

Characteristics		All	Type		*p*-value*
			non-IBC	IBC	
Median age, years (range)		54.5 (15–93.76)	58.29 (24.55–93.76)	49 (15–81)	**6.06E-10**
TNM, N					**3.53E-22**
	0	287 (59%)	261 (71%)	26 (21%)	
	1,2,3	200 (41%)	105 (29%)	95 (79%)	
TNM, M					**4.16E-15**
	0	458 (94%)	364 (99%)	94 (78%)	
	1	28 (6%)	2 (1%)	26 (22%)	
Pathological type					**1.20E-05**
	ductal	369 (75%)	256 (70%)	113 (90%)	
	lobular	56 (11%)	48 (13%)	8 (6%)	
	mixed	17 (3%)	17 (5%)	0 (0%)	
	other	49 (10%)	45 (12%)	4 (3%)	
Pathological grade					**< 1.00E-06**
	1	127 (26%)	119 (33%)	8 (7%)	
	2	188 (39%)	149 (41%)	39 (33%)	
	3	168 (35%)	97 (27%)	71 (60%)	
Pathological tumor size, pT					
	pT1	147 (41%)	147 (41%)	NR	
	pT2	149 (42%)	149 (42%)	NR	
	pT3	59 (17%)	59 (17%)	NR	
Pathological axillary node status, pN					
	negative	177 (50%)	177 (50%)	NR	
	positive	175 (50%)	175 (50%)	NR	
ER IHC status					**5.70E-04**
	negative	130 (27%)	82 (22%)	48 (39%)	
	positive	359 (73%)	284 (78%)	75 (61%)	
PR IHC status					**1.26E-02**
	negative	179 (37%)	122 (33%)	57 (46%)	
	positive	310 (63%)	244 (67%)	66 (54%)	
HER2 IHC status					**2.07E-17**
	negative	346 (83%)	295 (92%)	51 (52%)	
	positive	72 (17%)	25 (8%)	47 (48%)	
Molecular subtype					**< 1.00E-06**
	TN	58 (14%)	44 (14%)	14 (14%)	
	HR−/HER2+	33 (8%)	12 (4%)	21 (21%)	
	HR+/HER2−	288 (69%)	251 (78%)	37 (38%)	
	HR+/HER2+	39 (9%)	13 (4%)	26 (27%)	

*, comparison IBC *versus* non-IBC; NR: not relevant.

### MARCKS protein expression in breast cancer

**Figure 1:**
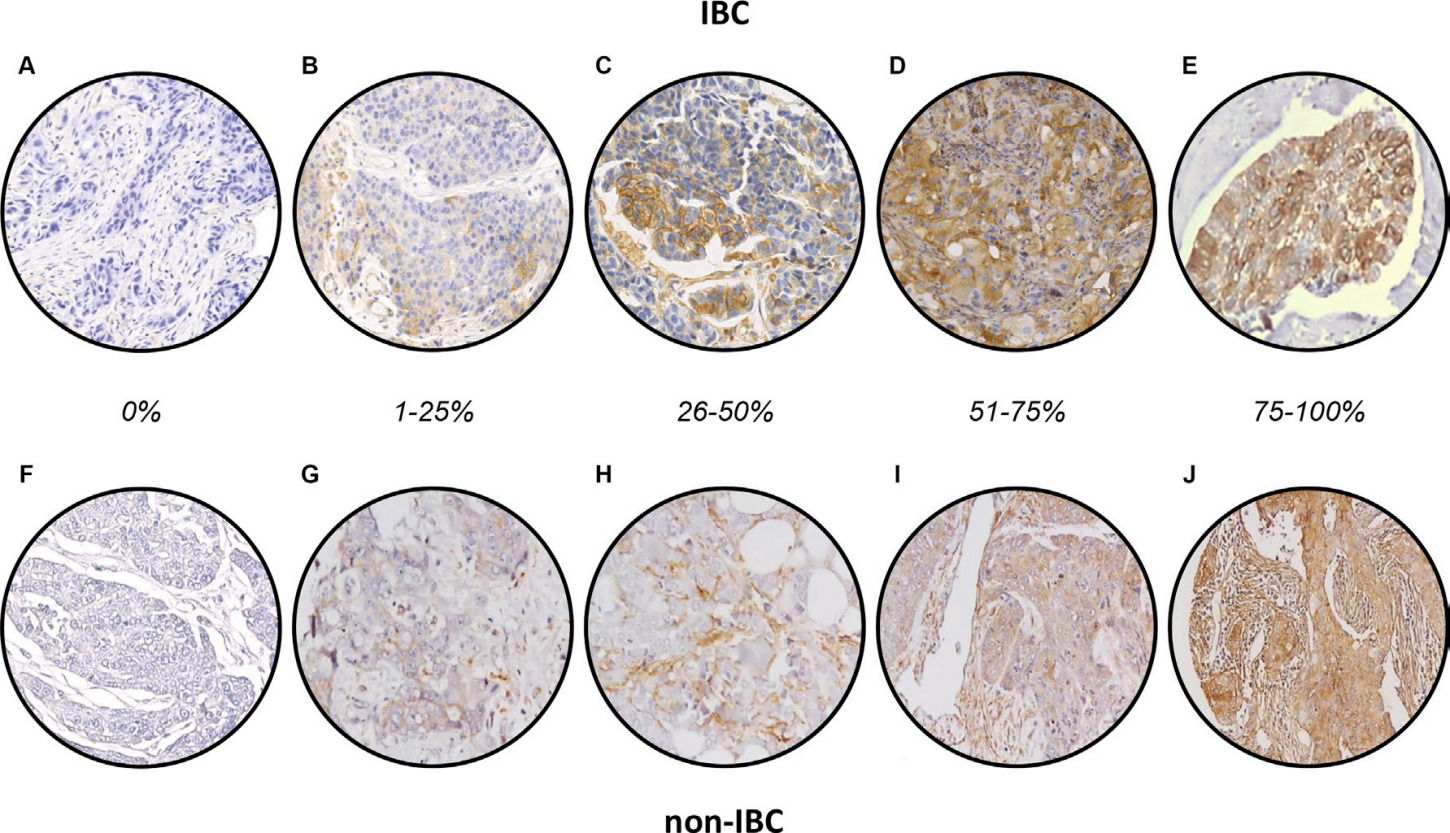
MARCKS immunostaining in breast cancer (**A**–**E**) Representative images of IHC staining in IBC samples showing different percentage of expression: 0% (A), 1–25% (B), 26–50% (C), 56–75% (D), and 76–100% (E). A shows the negative membranous expression, whereas E shows strong cytoplasmic and membranous expression in tumor emboli. F-J/ Representative images of IHC staining in non-IBC samples: F show the negative membranous expression. F-J/ Representative images of IHC staining in non-IBC samples showing different percentage of expression: 0% (**F**), 1–25% (**G**), 26–50% (**H**), 56–75% (**I**), and 76–100% (**J**). F shows the negative membranous expression.

Before analysis of tissue samples, we validated the MARCKS antibody using western blot analysis on three breast cancer cell lines with known mRNA expression. As shown in Supplementary Figure S1, the antibody specifically recognized MARCKS protein with a good correlation between protein and mRNA expression levels. MARCKS expression was then measured on the 502 tumor samples. Examples of staining are shown in Figure 1. The staining was observed mainly in tumor cells and to a lesser degree in stroma, notably fibroblasts, whereas it was only weakly expressed in normal epithelial cells. Tumor cell staining was mainly located in the cytoplasm, but, in some samples, some staining was observed on the cytoplasmic membrane. No correlation was established between this membrane staining and clinical features. There was high heterogeneity between breast cancers with respect to MARCKS staining (Table 2). The percentage of positively stained tumor cells ranged from 0% to 100%, with a median of 0%; 82% of samples showed 0% of stained tumor cells, 7% showed 1 to 25% of stained tumor cells, 3% showed 26 to 50% of stained tumor cells and 51 to 75% of stained tumor cells, and 5% showed 76 to 100% of stained tumor cells. Examples of staining are shown in Figure 1. In IBC samples, MARCKS staining was observed in some cases within the dermal tumor emboli (Figure 1E). Using 1% of stained tumor cells as positivity cut-off, 89 samples (18%) exhibited a positive MARCKS expression (≥ 1% of stained cells), whereas 413 (82%) were MARCKS-negative. The MARCKS staining intensity was also heterogeneous between all samples, ranging from null to strong, with a null median intensity; the intensity was null in 87% of cases, low in 6%, moderate in 5%, and strong in 2%. Among the 502 samples tested, 148 samples (34 IBC and 114 non-IBC) were previously analyzed for *MARCKS* mRNA expression on DNA microarrays [7]: as shown in Supplementary Figure S2, there was a correlation between protein and mRNA expression (*p* = 7.0E-03).

**Table 2:** MARCKS IHC results in all breast cancer samples, in IBC and non-IBC

MARCKS IHC variable		All BC (*N* = 502)	non-IBC (*N* = 369)	IBC (*N* = 133)	Odds Ratio [95 CI]	*p*-value**
Percentage of positive tumor cells	median (range)	0 (0–100)	0 (0–90)	0 (0–100)		**1.55E-10**
	0%	413 (82%)	328 (89%)	85 (64%)		**1.00E-06**
	1–25%	37 (7%)	16 (4%)	21 (16%)		
	26–50%	14 (3%)	5 (1%)	9 (7%)		
	51–75%	16 (3%)	7 (2%)	9 (7%)		
	76–100%	22 (5%)	13 (4%)	9 (7%)		
	negative (0%)	413 (82%)	328 (89%)	85 (64%)	4.5 [2.71–7.51]	**1.42E-09**
	positive (1–100%)	89 (18%)	41 (11%)	48 (36%)		
Staining intensity*	median (range)	0 (0–3)	0 (0–3)	0 (0–3)		**9.63E-03**
	null	413 (87%)	328 (89%)	85 (79%)		**4.93E-02**
	low	30 (6%)	21 (6%)	9 (8%)		
	moderate	25 (5%)	15 (4%)	10 (9%)		
	strong	8 (2%)	5 (1%)	3 (3%)		
	negative (null)	413 (87%)	328 (89%)	85 (79%)	2.1 [1.11–3.77]	**1.48E-02**
	positive (low to strong)	63 (13%)	41 (11%)	22 (21%)		

*N = 476; **p-value for the IBC *vs* non-IBC comparison.

### MARCKS protein expression and clinicopathological features

We searched for correlations between binary MARCKS IHC status and clinicopathological features of samples. As shown in Table 3, no correlation was found with patients’ age, pathological type, pathological tumor size, and pathological axillary lymph node status. Conversely, correlation was observed with other features: MARCKS-positivity was more frequently associated with clinical axillary lymph node positive status (*p* = 8.1E-03), metastatic stage (*p* = 4.0E-02), high grade (*p* = 5.0E-06), ER-negativity (*p* = 1.6E-08), PR-negativity (*p* = 6.4E-08), HER2-positivity (*p* = 1.5E-04), and triple-negative and HER2+ statutes (*p* < 1.0E-06). Importantly, MARCKS expression was more frequently positive in IBC than in non-IBC (*p* = 1.4E-09; Supplementary Figure S3A). Fifty-four percent of MARCKS-positive samples were IBC *versus* only 21% of MARCKS-negative samples (OR = 4.5; [95 CI, 2.7–7.5]). The percentage of MARCKS-positive cases was 36% in IBC *versus* 11% in non-IBC (Table 2), and was not different between the Tunisian and French IBC samples (42 *versus* 30%, *p* = 0.21; Supplementary Figure S3B). As shown in Table 2, the percentage of positively stained tumor cells, analyzed as continuous value and as discrete value in two or five subgroups) was significantly higher in IBC than in non-IBC. Similarly, the MARCKS staining intensity was significantly higher in IBC than in non-IBC, both as continuous and discrete values.

**Table 3:** Clinicopathological correlations with MARCKS expression

Characteristics		MARCKS IHC			*p*-value
		*N*	negative	positive	
Median age, years (range)		494	54.88 (24.55−93.76)	54 (15−86.56)	0.136
TNM, N					**8.07E-03**
	0	287	247 (62%)	40 (46%)	
	1,2,3	200	153 (38%)	47 (54%)	
TNM, M					**4.05E-02**
	0	458	383 (95%)	75 (89%)	
	1	28	19 (5%)	9 (11%)	
Pathological type					0.175
	ductal	369	298 (74%)	71 (82%)	
	lobular	56	48 (12%)	8 (9%)	
	mixed	17	17 (4%)	0 (0%)	
	other	49	41 (10%)	8 (9%)	
Pathological grade					**5.00E-06**
	1	127	116 (29%)	11 (13%)	
	2	188	164 (41%)	24 (29%)	
	3	168	119 (30%)	49 (58%)	
Pathological tumor size, pT					0.204
	pT1	147	132 (42%)	15 (34%)	
	pT2	151	127 (40%)	24 (55%)	
	pT3	61	56 (18%)	5 (11%)	
Pathological axillary node status, pN					0.637
	negative	184	163 (51%)	21 (46%)	
	positive	184	159 (49%)	25 (54%)	
ER IHC status					**1.58E-08**
	negative	130	85 (21%)	45 (52%)	
	positive	359	318 (79%)	41 (48%)	
PR IHC status					**6.37E-08**
	negative	179	125 (31%)	54 (63%)	
	positive	310	278 (69%)	32 (37%)	
ERBB2 IHC status					**1.47E-04**
	negative	346	296 (86%)	50 (67%)	
	positive	72	47 (14%)	25 (33%)	
Molecular subtype					**< 1.00E-06**
	TN	58	37 (11%)	21 (28%)	
	HR−/HER2+	33	19 (6%)	14 (19%)	
	HR+/HER2−	288	259 (76%)	29 (39%)	
	HR+/HER2+	39	28 (8%)	11 (15%)	
Type					**1.42E-09**
	non-IBC	369	328 (79%)	41 (46%)	
	IBC	133	85 (21%)	48 (54%)	

### Uni- and multivariate analyses of the IBC *versus* non-IBC distinction

Thus, MARCKS-positivity was associated with IBC phenotype. Because of association with other clinicopathological features, themselves known to be associated with the IBC/non-IBC distinction, we did uni- and multivariate analyses centered on the comparison IBC *versus* non-IBC (Table 4). In univariate analysis, as expected all tested variables (patients’ age, clinical lymph node and metastatic extension, pathological type, grade, molecular subtypes, and MARCKS status) could distinguish IBC and non-IBC statutes. In multivariate analysis integrating all significant variables, MARCKS protein expression remained associated with the IBC phenotype (*p* = 6.9E-04), suggesting discriminating value independent from other variables including the molecular subtypes.

**Table 4:** Uni- and multivariate analyses of IBC/non-IBC distinction

Characteristics		Univariate			Multivariate		
		*N*	OR [95 CI]	*p*-value	*N*	OR [95 CI]	*p*-value
Age, years		494	0.95 [0.93–0.96]	**2.68E-09**	405	0.95 [0.93–0.97]	**4.92E-04**
TNM, N	1,2,3 vs. 0	487	9.08 [6.02–13.7]	**9.88E-19**	405	10.2 [5.24–19.7]	**8.32E-09**
TNM, M	1 vs. 0	486	50.3 [14.83–171]	**1.33E-07**	405	3.9E8 [0.00–Inf]	0.988
Pathological type	lobular vs. ductal	491	0.38 [0.20–0.73]	**1.45E-02**	405	1.05 [0.33–3.39]	0.941
	mixed vs. ductal	491	0 [0–Inf]	0.978	405	0 [0–Inf]	0.990
	other vs. ductal	491	0.20 [0.08–0.48]	**2.68E-03**	405	0.25 [0.07–0.90]	0.075
Pathological grade	2 vs. 1	483	3.89 [1.99–7.61]	**8.42E-04**	405	1.61 [0.57–4.57]	0.450
	3 vs. 1	483	10.9 [5.66–20.9]	**1.85E-09**	405	4.25 [1.53–11.8]	**2.02E-02**
Molecular subtype	HR-/HER2+ vs. HR+/HER2−	418	11.9 [6.12–23.1]	**7.86E-10**	405	0.98 [0.42–2.31]	0.975
	HR+/HER2+ vs. HR+/HER2−	418	13.6 [7.23–25.5]	**9.40E-12**	405	2.44 [0.98–6.06]	0.107
	TN vs. HR+/HER2−	418	2.16 [1.21–3.86]	**2.96E-02**	405	8.93 [3.58–22.3]	**8.32E-05**
MARCKS IHC status	positive vs. negative	502	4.52 [3.02–6.76]	**7.53E-10**	405	4.42 [2.15–9.09]	**6.99E-04**

### Uni- and multivariate prognostic analyses for MFS

**Figure 2:**
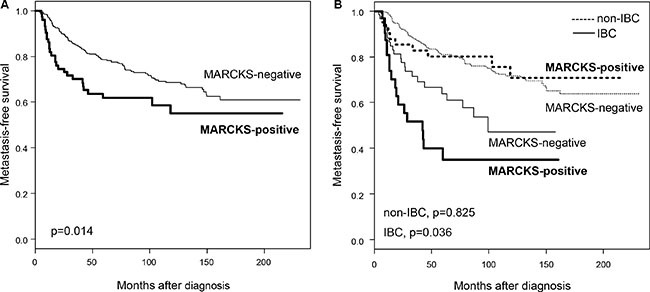
Metastasis-free survival according to MARCKS expression in the whole population and in IBC and non-IBC (**A**) Kaplan-Meier MFS curves in patients with negative and positive expression in the whole population. (**B**) Similar to (A), but in patients with non-IBC (dashed lines) and patients with IBC (full lines). The *p*-values of log-rank test are indicated.

We assessed the prognostic value of MARCKS expression in terms of MFS. MFS data were available for 458 non-metastatic (M0) patients, including 320 who remained metastasis-free during a median follow-up of 99 months (range, 5 to 231) and 138 who displayed metastatic relapse. The 5-year MFS rate was 76% [95 CI, 72–80]. In univariate analysis (Table 5), pathological type, clinical lymph node extension, high grade, IBC phenotype, and MARCKS expression (*p* = 1.5E-02, Wald test; HR = 1.67 [95 CI, 1.10–2.53]) were associated with poor MFS. The 5-year MFS rate was 62% [95 CI, 51–75] in the MARCKS-positive group *versus* 78% [95CI, 74–83] in the MARCKS-negative group (*p* = 1.4E-02, log-rank test; Figure 2A). In multivariate analysis (Table 5), pathological type, clinical lymph node extension, grade, and IBC phenotype remained significant, whereas MARCKS expression lost its prognostic value (*p* = 0.163, Wald test; HR = 1.36 [95 CI, 0.88–2.10]).

**Table 5:** Uni- and multivariate prognostic analyses for MFS

	Characteristics		Univariate			Multivariate		
			*N*	HR [95 CI]	*p*-value	*N*	HR [95 CI]	*p*-value
**All breast cancers**	Age, years		458	0.99 [0.98–1.01]	0.24			
	Pathological type	lobular vs. ductal	458	1.16 [0.72–1.88]	**2.37E-02**	452	1.70 [1.03–2.8]	**3.69E-02**
		mixed vs. ductal		1.13 [0.52–2.42]		452	1.49 [0.68–3.27]	0.324
		other vs. ductal		0.22 [0.08–0.61]		452	0.30 [0.11–0.83]	**2.05E-02**
	TNM, N	1,2,3 vs. 0	457	2.52 [1.80–3.52]	**7.88E-08**	452	2.08 [1.46–2.98]	**5.63E-05**
	Pathological grade	2 vs. 1	453	2.03 [1.24–3.33]	**2.33E-06**	452	1.69 [1.02–2.82]	**4.34E-02**
		3 vs. 1		3.44 [2.11–5.60]		452	2.44 [1.45–4.11]	**8.08E-04**
	Molecular subtype	HR−/HER2+ vs. TN	394	1.46 [0.64–3.30]	0.161			
		HR+/HER2− vs. TN		0.79 [0.46–1.35]				
		HR+/HER2+ vs. TN		1.29 [0.61–2.73]				
	Type	IBC vs. non-IBC	458	2.97 [2.04–4.31]	**1.13E-08**	452	1.96 [1.29–2.98]	**1.63E-03**
	MARCKS IHC status	positive vs. negative	458	1.67 [1.10–2.53]	**1.56E-02**	452	1.36 [0.88–2.10]	0.163
**IBC**	Age, years		94	1.02 [1.00–1.05]	0.059			
	Pathological type	lobular vs. ductal	94	1.13 [0.35–3.69]	0.666			
		mixed vs. ductal		NA [ NA – NA]				
		other vs. ductal		0.41 [0.06–3.03]				
	TNM, N	1,2,3 vs. 0	93	1.66 [0.79–3.53]	0.184			
	Pathological grade	2 vs. 1	90	0.62 [0.18–2.05]	0.31			
		3 vs. 1		1.14 [0.40–3.25]				
	Molecular subtype	HR−/HER2+ vs. TN	74	0.88 [0.25–3.07]	0.961			
		HR+/HER2− vs. TN		0.76 [0.27–2.16]				
		HR+/HER2+ vs. TN		0.81 [0.27–2.43]				
	MARCKS IHC status	positive vs. negative	94	1.92 [1.03–3.59]	**3.95E-02**	94	1.92 [1.03–3.59]	**3.95E-02**
**non-IBC**	Age, years		364	0.99 [0.98–1.01]	0.506			
	Pathological type	lobular vs. ductal	364	1.39 [0.82–2.36]	**2.83E-02**	351	1.68 [0.97–2.93]	0.064
		mixed vs. ductal		1.46 [0.67–3.17]		351	1.52 [0.69–3.34]	0.298
		other vs. ductal		0.23 [0.07–0.74]		351	0.35 [0.11–1.12]	0.078
	Pathological axillary node status, pN	positive vs. negative	352	3.08 [1.95–4.87]	**1.46E-06**	351	2.25 [1.4–3.63]	**8.246E-04**
	Pathological tumor size, pT	pT2 vs. pT1	355	2.71 [1.62–4.53]	**1.50E-05**	351	1.78 [1.03–3.07]	**3.724E-02**
		pT3 vs. pT1		3.83 [2.14–6.85]		351	2.51 [1.38–4.56]	**2.593E-03**
	Pathological grade	2 vs. 1	363	2.25 [1.30–3.88]	**7.45E-04**	351	1.62 [0.91–2.88]	0.100
		3 vs. 1		3.02 [1.70–5.37]		351	2.38 [1.27–4.44]	**6.714E-03**
	Molecular subtype	HR−/HER2+ vs. TN	320	1.27 [0.40–3.99]	0.909			
		HR+/HER2− vs. TN		0.91 [0.48–1.72]				
		HR+/HER2+ vs. TN		0.80 [0.22–2.88]				
	MARCKS IHC status	positive vs. negative	364	0.93 [0.48–1.79]	0.825			

The same analysis was then done in each clinical type separately, IBC and non-IBC (Table 5). In non-IBC (*N* = 364), the 5-year MFS rate was 81% (95 CI, 77–85) with 97 metastatic events, and the median follow-up was 111 months (range, 5 to 231). In univariate analysis, pathological type, tumor size, axillary lymph node status and grade were associated with MFS, whereas MARCKS expression was not (*p* = 0.825, Wald test; HR = 0.93 [95 CI, 0.48–1.79]; Figure 2B). By contrast, in IBC (*N* = 94), MARCKS expression was associated with poor MFS (*p* = 3.9E-02, Wald test; HR = 1.93 [95 CI, 1.03–3.59], whereas the other tested prognostic factors were not. The 5-year MFS rate was 35% [95 CI, 20–59] in the MARCKS-positive group *versus* 64% [95 CI, 52–79] in the MARCKS-negative group (*p* = 3.6E-02, log-rank test; Figure 2B).

## DISCUSSION

Despite therapeutic progresses achieved during the last decades, the survival of patients with IBC remains unfavorable, and the identification of new therapeutic targets is crucial. Our objective was to validate at the protein level the overexpression of MARCKS in IBC in a large series of clinical samples (133 IBC and 369 non-IBC) and to search for correlations with tumor features. We showed that MARCKS expression was more frequent in IBC than non-IBC and was associated with poor-prognosis features in the whole series of samples. In IBC, MARCKS expression was associated with poor MFS.

We focused on MARCKS protein expression for several reasons: i) strong differential mRNA expression between IBC and non-IBC clinical samples in the largest transcriptomics study reported to date and stratified upon molecular subtypes [7]; ii) proven role of MARCKS in cancer progression including metastatic process and therapeutic resistance; iii) ongoing development of MARCKS inhibitors; iv) commercial availability of a corresponding monoclonal antibody performing sufficiently well in IHC on paraffin-embedded tissues as previously reported [18, 21]. Before analysis of tissue samples, we revalidated the antibody on breast cancer cell lines by using western blot analysis.

We found heterogeneous MARCKS staining between all breast cancers. Eighteen percent of cases were scored as MARCKS-positive and 82% as MARCKS-negative. In the literature, only one study analyzed MARCKS protein expression in breast cancer using IHC in a series of 250 non-IBC spotted onto tissue microarrays [21]: MARCKS was first identified using proteomics as the fourth most upregulated protein in tamoxifen-resistant MCF7 breast cancer cells as compared to the MCF7 parental cell line. Then, the knockdown of MARCKS in tamoxifen-resistant MCF7 cells was shown to decrease cell motility, whereas analysis of a panel of 21 breast cancer cell lines showed correlation of MARCKS mRNA and protein expression, as we observed here in our panel of three breast cancer cell lines and 148 clinical samples. Finally, IHC analysis with the same antibody and positivity cut-off (1% cut-off) as us in the present study showed a MARCKS-positive cytoplasmic staining in 40% of samples. The relative lower frequency of positivity that we found (18%) may be explained notably by the imperfect reproducibility of IHC and the heterogeneity of breast cancer. The same study also reported positive correlations between MARCKS-positivity and poor-prognosis features: metastatic stage, high grade, ER-negativity, PR-negativity, HER2-positivity, triple-negative and HER2+ statutes, as we found here in our larger series. Additionally, the authors showed positive correlation of MARCK expression with poor specific overall survival in all cases and in ER-positive cases in uni- and multivariate analyses. In our larger series, we confirmed the poor-prognosis value of MARCKS expression in breast cancer in univariate but not in multivariate analysis. The other study on MARCKS and breast cancer was recently published [22]. In small series of 21 and 50 breast tumors, including a majority of cancers, the authors showed frequent high IHC staining for phospho-MARCKS in breast cancer as compared to adjacent normal breast tissue and correlation with poor differentiation/high grade and metastatic status. Using both *in vitro* and *in vivo* models of triple-negative breast cancers, they then showed accumulation of phospho-MARCKS in response to paclitaxel treatment and an increased paclitaxel sensitivity after reduction of phospho-MARCKS by knockdown or by treatment with MANS peptide, a phospho-MARCKS inhibitor targeting the N-terminal myristoylation site. The MANS peptide also attenuated angiogenesis/metastasis of xenografted breast cancer cells. More recently, the unfavorable prognostic impact of high *MARCKS* mRNA expression on patients’ survival was found in a large annotated database of breast cancer patient samples [18]. Altogether, these studies suggested critical roles of MARCKS in the regulation of breast cancer aggressiveness, and therapeutic resistance, notably to hormone therapy and chemotherapy. In this context, the overexpression of MARCKS in IBC *versus* non-IBC seems coherent given the classical metastatic propensity and resistance of IBC. Interestingly, our multivariate analysis showed that the discriminating value of MARCKS expression between both phenotypes was independent from all other discriminating variables including the molecular subtypes (as previously observed at the mRNA level [7]) and the disease extension, suggesting that MARCKS expression *per se* represents a new discriminating feature. One possible mechanism of MARCKS overexpression in IBC might be the downregulation of miR-30b, a phosphoinositide 3-kinase (PI3K)-targeting miRNA, which targets notably MARCKS [23]. Molecular profiling of experimental models with and without MARCKS activation could provide relevant insight about the regulation and the consequences of MARCKS expression in breast cancer. A more indirect way is to analyze gene expression profiles of clinical tumor samples according to MARCKS protein expression. From the 148 samples of our series for which gene expression data were also available, only 27 were MARCKS-positive, thus impeding any robust supervised analysis. We applied gene set enrichment analysis (GSEA) algorithm [24] to these 148 expression profiles and the 674 Reactome gene sets (C2) of the MSigDB database. Analysis identified several significant gene sets (1000 gene sets permutations, *p* < 0.05, FDR < 0.25) involved in cell proliferation, cell adhesion and immune response (Supplementary Figure S4). Interestingly, the unfavorable prognostic value of MARCKS expression for MFS was limited to IBC.

Several studies in other solid cancers have suggested the involvement of MARCKS in tumor progression and resistance, and its interest as novel therapeutic target. By employing *in vitro* and *in vivo* approaches, Rombouts et al. showed an articulated role for MARCKS in the progression of colorectal cancer and suggested a suitable target to interfere and overcome the invasive behavior of colon carcinoma cells at primary and distant sites [15]. In melanoma, Chen et al. demonstrated that phospho-MARCKS contributed to the metastatic potential of melanoma cells [13]. In cholangiocarcinoma, Techassen et al. showed in a series of 60 clinical samples that patients with high MARCKS expression had shorter survival than patients with low expression. Then, using experimental models, they reported MARCKS as one of the key players in the migration of cholangiocarcinoma cells and suggested that the cycling between MARCKS and phospho-MARCKS might regulate the metastasis of biliary cancer cells [16]. In ovarian carcinoma, *in silico* analysis of a large transcriptomic database showed that high *MARCKS* mRNA expression was associated with poor patient survival [18]. In a series of 10 normal ovarian tissues and 18 pairs of primary and metastatic epithelial ovarian cancer tissues analyzed by IHC, MARCKS protein was highly expressed in ovarian tumor stroma gradually as cancer progressed, and was required for the differentiation and tumor-promoting function of cancer-associated fibroblasts, including proliferation, chemotherapeutical resistance and migration *in vitro*. Analysis of MARCKS stromal staining intensity in our present series (data not shown) showed a moderate to strong expression in fibroblasts more frequent in IBC (31% of the samples) than in non-IBC (24% of the samples; *p* = 0.009, Fisher's exact test), which might contribute to more cancer-associated fibroblasts activation in IBC and higher metastatic potential. In non-small-cell lung (NSCL) cancer, several studies suggested an oncogenic role for MARCKS. In a series of 99 patients with squamous cell carcinoma [14], a significant association was reported between positive IHC expression and poor survival. In another study [20], elevated levels of MARCKS and phospho-MARCKS were found in highly invasive lung cancer cell lines and clinical NSCL cancer samples. MARCKS knockdown reduced cell migration of highly invasive cancer cell lines and suppressed PI3K (phosphatidylinositol 3′-kinase)/AKT phosphorylation and SLUG level. Finally, treatment with the MANS peptide impaired cell migration *in vitro* and the metastatic potential of invasive lung cancer cells *in vivo*, through coordination of increase of E-cadherin expression, suppression of MARCKS phosphorylation and AKT/SLUG signaling pathway. In a second study [19], higher phospho-MARCKS staining was associated with shorter survival in a series of 195 operated lung cancers and associated with EGFR-TKI-based treatment in a series of 52 treated metastatic patients. *In vitro* models showed that phospho-MARCKS promoted cancer growth and erlotinib resistance. Treatment with a 25-mer peptide targeting the MARCKS phosphorylation site domain (MPS peptide) suppressed tumor growth and metastasis *in vivo*, reduced levels of phospho-MARCKS and PIP3, and enhanced the sensitivity of lung cancer cells to erlotinib treatment *in vitro* and *in vivo*. These results indicated a crucial role for MARCKS, specifically its phosphorylated form, in potentiating lung cancer cell migration/metastasis/growth and suggested a potential use of MARCKS-related peptides in the treatment of lung cancer metastasis. Thus, our results (overexpression of MARCKS in IBC *versus* non-IBC, association with poor-prognosis variables in breast cancers, unfavorable independent prognostic value in IBC) are consistent with the clinical and pre-clinical findings published in breast cancer and other cancers (colorectal, ovarian, lung, cholangiocarcinoma, melanoma).

In conclusion, we showed a significant MARCKS protein overexpression in IBC when compared with non-IBC, independent from other clinicopathological variables, and an association with poor MFS in IBC. The strengths of our study include the number of cases tested with a total of 502 breast cancers including 133 IBC, a tumor rare but aggressive; to our knowledge, it is the first study analyzing specifically MARCKS protein expression in IBC and the largest one in breast cancer. Our study provides a first independent validation of one of the key genes in the IBC-specific 79-gene signature [7] and therefore lends credit to the signature as a whole to properly reflect IBC biology. Limitations include the retrospective nature and associated biases such as missing data with the absence of survival information for all patients. But yet, our results suggest that MARCKS overexpression might in part explain the poor prognosis of IBC and that MARCKS, as an oncogene associated with poor MFS in IBC, might represent a new potential target for therapeutic intervention. In this context, the assessment of MARCKS-related peptides such as MANS and MPS peptides in the treatment of IBC pre-clinical models is urgently warranted. The analysis of phospho-MARCKS expression in our series is another relevant objective that might not only improve the discrimination between IBC and non-IBC when compared to MARCKS expression, but also improve the prognostic value and help predicting the response to MARCKS-related peptides.

## MATERIALS AND METHODS

### Patients and samples

In this retrospective study, we collected pre-therapeutic diagnostic tumor samples from 133 patients with IBC treated at Institut Paoli-Calmettes (IPC) of Marseille (France; *N* = 69) and Institute Salah Azaiez of Tunis (Tunisia; *N* = 64). Main inclusion criteria were female patient, with clinically-defined and pathologically-confirmed IBC (AJCC T4d), written informed consent, available formaldehyde-fixed and paraffin-embedded pre-therapeutic diagnostic tumor sample and clinicopathological annotations. MARCKS immunohistochemistry (IHC) was done on standard slides. The study was approved by institutional review boards of the two participating centers. The control group included pre-therapeutic non-IBC samples from 369 women treated at IPC for a pathologically-confirmed invasive breast carcinoma, representing a mixture of early and advanced stages. Two tissue microarrays (TMA) were available for those 369 cases. They were constructed as previously described, with slight modifications [25]. For each sample, three representative tumor areas were carefully selected from a hematoxylin-eosin stained section of the donor block. Core cylinders with a diameter of 0.6 mm each were punched from each of these areas and deposited into the recipient paraffin block using a specific arraying device (Alphelys, Plaisir, France). Five-μm sections of the resulting TMA blocks were made and used for IHC. Clinicopathological annotations included patients’ age at diagnosis, TNM stage, pathological features (type, grade, tumor size and axillary lymph node status), ER, PR and HER2 IHC statutes, and clinical outcome. The molecular subtypes of tumors were based on ER, PR and HER2 IHC statutes and included four categories: HR+/HER2−, HR+/HER2+, HR−/HER2+, and HR−/HER2−.

### Immunohistochemical analysis

MARCKS protein expression was analyzed on standard slides for the 133 IBC and TMAs for the 369 non-IBC using standard IHC protocols. IHC was performed on 4-μm sections. Paraffin sections were pretreated in PT Link PH6 (DakoCytomation, Copenhagen, Denmark). MARCKS staining was done with the rabbit monoclonal antibody, anti-MARCKS (D88D11) XP^®^Rabbit mAb#5607, from Cell Signaling Technology that was diluted at 1/400. We first validated the antibody using western blot analysis with breast cancer cell lines. Expression was analyzed in three breast cancer cell lines (T47D, SUM149, MDA-MB-231) previously profiled using Affymetrix DNA microarrays and for which *MARCKS* mRNA expression was documented as very low (T47D), moderate (SUM149), and very high (MDA-MB-231). Cells were washed 3 times with ice-cold PBS and then resuspended for 30 min in 750 μl of ice cold lysis buffer containing 50 mM Hepes, pH 7.5, 150 mM NaCl, 1.5 mM MgCl_2_, 1 mM EGTA, 1% Triton X-100, and 10% glycerol. A protease inhibitor mixture (Pierce) and the phosphotyrosyl phosphatase inhibitor sodium orthovanadate (BioLabs) were added as recommended. Lysates were heated in SDS sample buffer (60 mM Tris-HCl, pH 6.7, 3% SDS, 2% (v/v) 2-mercaptoethanol, and 5% glycerol), separated by 10% SDS-PAGE, and transferred to nitrocellulose blotting membrane (Amersham). Membranes were blocked in PBS supplemented with BSA 5% and tween 0.1% for 1 h30 and then incubated overnight at 4°C with indicated antibodies. Visualization was done with ECL (Pierce). For the IHC analysis, the antigen was revealed by the Dako Flex system (Dako) using a peroxidase enzyme. Sections counterstained with hematoxylin were independently evaluated by two experienced breast pathologists (JTP and JJ) using light microscopy. Immunoreactivities were scored mainly by measuring the percentage of positive tumor cells, from 0% for undetectable level to 100% for total homogeneous staining. MARCKS-negative cases were defined by 0% level and positive cases by at least 1% of stained tumor cells.

### Statistical analysis

Data were summarized by numbers and percentages for categorical variables, and median and range for continuous variables. Correlations between tumor groups and clinicopathological features were analyzed using the *t*-test or the Fisher's exact test when appropriate. Follow-up was calculated from the date of diagnosis to the date of last news for event-free patients. Metastasis-free survival (MFS) was calculated from the date of diagnosis until the date of first distant relapse. Survival was calculated using the Kaplan-Meier method and curves were compared with the log-rank test. Uni- and multivariate analyses regarding the IBC/non-IBC distinction was done using logistic regression analysis using the glm function in R's statistical package and the significance was estimated by specifying a binomial family for model with a logit link. Uni- and multivariate prognostic analyses for MFS were done using Cox regression analysis (Wald test). Variables with a *p*-value < 0.05 in univariate analysis were tested in multivariate analysis. All statistical tests were two-sided at the 5% level of significance. Analyses were done using the survival package (version 2.30) in the R software (version 2.9.1; http://www.cran.r-project.org/). We followed the reporting REcommendations for tumor MARKer prognostic studies (REMARK criteria) [26].

## SUPPLEMENTARY MATERIALS FIGURES



## References

[1] Dawood S, Merajver SD, Viens P, Vermeulen PB, Swain SM, Buchholz TA, Dirix LY, Levine PH, Lucci A, Krishnamurthy S, Robertson FM, Woodward WA, Yang WT (2011). International expert panel on inflammatory breast cancer: consensus statement for standardized diagnosis and treatment. Ann Oncol.

[2] Boussen H, Bouzaiene H, Ben Hassouna J, Dhiab T, Khomsi F, Benna F, Gamoudi A, Mourali N, Hechiche M, Rahal K, Levine PH (2010). Inflammatory breast cancer in Tunisia: epidemiological and clinical trends. Cancer.

[3] Charafe-Jauffret E, Tarpin C, Viens P, Bertucci F (2008). Defining the molecular biology of inflammatory breast cancer. Semin Oncol.

[4] Bertucci F, Finetti P, Birnbaum D, Viens P (2010). Gene expression profiling of inflammatory breast cancer. Cancer.

[5] Bekhouche I, Finetti P, Adelaide J, Ferrari A, Tarpin C, Charafe-Jauffret E, Charpin C, Houvenaeghel G, Jacquemier J, Bidaut G, Birnbaum D, Viens P, Chaffanet M (2011). High-resolution comparative genomic hybridization of inflammatory breast cancer and identification of candidate genes. PLoS One.

[6] Bertucci F, Finetti P, Vermeulen P, Van Dam P, Dirix L, Birnbaum D, Viens P, Van Laere S (2014). Genomic profiling of inflammatory breast cancer: a review. Breast.

[7] Van Laere S, Ueno NT, Finetti P, Vermeulen PB, Lucci A, Robertson F, Marsan M, Iwamoto T, Krishnamurthy S, Masuda H, van Dam P, Woodward WA, Viens P (2013). Uncovering the molecular secrets of Inflammatory Breast Cancer biology: An integrated analysis of three distinct Affymetrix gene expression data sets. Clin Cancer Res.

[8] Ouimet CC, Wang JK, Walaas SI, Albert KA, Greengard P (1990). Localization of the MARCKS (87 kDa) protein, a major specific substrate for protein kinase C, in rat brain. J Neurosci.

[9] Aderem A (1992). The MARCKS brothers: a family of protein kinase C substrates. Cell.

[10] Ohmitsu M, Fukunaga K, Yamamoto H, Miyamoto E (1999). Phosphorylation of myristoylated alanine-rich protein kinase C substrate by mitogen-activated protein kinase in cultured rat hippocampal neurons following stimulation of glutamate receptors. J Biol Chem.

[11] Tatsumi S, Mabuchi T, Katano T, Matsumura S, Abe T, Hidaka H, Suzuki M, Sasaki Y, Minami T, Ito S (2005). Involvement of Rho-kinase in inflammatory and neuropathic pain through phosphorylation of myristoylated alanine-rich C-kinase substrate (MARCKS). Neuroscience.

[12] Rath N, Olson MF (2012). Rho-associated kinases in tumorigenesis: re-considering ROCK inhibition for cancer therapy. EMBO Rep.

[13] Chen X, Rotenberg SA (2010). PhosphoMARCKS drives motility of mouse melanoma cells. Cell Signal.

[14] Hanada S, Kakehashi A, Nishiyama N, Wei M, Yamano S, Chung K, Komatsu H, Inoue H, Suehiro S, Wanibuchi H (2013). Myristoylated alanine-rich C-kinase substrate as a prognostic biomarker in human primary lung squamous cell carcinoma. Cancer Biomark.

[15] Rombouts K, Carloni V, Mello T, Omenetti S, Galastri S, Madiai S, Galli A, Pinzani M (2013). Myristoylated Alanine-Rich protein Kinase C Substrate (MARCKS) expression modulates the metastatic phenotype in human and murine colon carcinoma in vitro and in vivo. Cancer Lett.

[16] Techasen A, Loilome W, Namwat N, Takahashi E, Sugihara E, Puapairoj A, Miwa M, Saya H, Yongvanit P (2010). Myristoylated alanine-rich C kinase substrate phosphorylation promotes cholangiocarcinoma cell migration and metastasis via the protein kinase C-dependent pathway. Cancer Sci.

[17] Yang Y, Chen Y, Saha MN, Chen J, Evans K, Qiu L, Reece D, Chen GA, Chang H (2015). Targeting phospho-MARCKS overcomes drug-resistance and induces antitumor activity in preclinical models of multiple myeloma. Leukemia.

[18] Yang Z, Xu S, Jin P, Yang X, Li X, Wan D, Zhang T, Long S, Wei X, Chen G, Meng L, Liu D, Fang Y (2016). MARCKS contributes to stromal cancer-associated fibroblast activation and facilitates ovarian cancer metastasis. Oncotarget.

[19] Chen CH, Statt S, Chiu CL, Thai P, Arif M, Adler KB, Wu R (2014). Targeting myristoylated alanine-rich C kinase substrate phosphorylation site domain in lung cancer. Mechanisms and therapeutic implications. Am J Respir Crit Care Med.

[20] Chen CH, Thai P, Yoneda K, Adler KB, Yang PC, Wu R (2014). A peptide that inhibits function of Myristoylated Alanine-Rich C Kinase Substrate (MARCKS) reduces lung cancer metastasis. Oncogene.

[21] Browne BC, Hochgrafe F, Wu J, Millar EK, Barraclough J, Stone A, McCloy RA, Lee CS, Roberts C, Ali NA, Boulghourjian A, Schmich F, Linding R (2013). Global characterization of signalling networks associated with tamoxifen resistance in breast cancer. FEBS J.

[22] Chen CH, Cheng CT, Yuan Y, Zhai J, Arif M, Fong LW, Wu R, Ann DK (2015). Elevated MARCKS phosphorylation contributes to unresponsiveness of breast cancer to paclitaxel treatment. Oncotarget.

[23] Van der Auwera I, Limane R, van Dam P, Vermeulen PB, Dirix LY, Van Laere SJ (2010). Integrated miRNA and mRNA expression profiling of the inflammatory breast cancer subtype. Br J Cancer.

[24] Subramanian A, Tamayo P, Mootha VK, Mukherjee S, Ebert BL, Gillette MA, Paulovich A, Pomeroy SL, Golub TR, Lander ES, Mesirov JP (2005). Gene set enrichment analysis: a knowledge-based approach for interpreting genome-wide expression profiles. Proc Natl Acad Sci USA.

[25] Ginestier C, Charafe-Jauffret E, Bertucci F, Eisinger F, Geneix J, Bechlian D, Conte N, Adelaide J, Toiron Y, Nguyen C, Viens P, Mozziconacci MJ, Houlgatte R (2002). Distinct and complementary information provided by use of tissue and DNA microarrays in the study of breast tumor markers. Am J Pathol.

[26] McShane LM, Altman DG, Sauerbrei W, Taube SE, Gion M, Clark GM (2005). Statistics Subcommittee of the NCIEWGoCD. REporting recommendations for tumour MARKer prognostic studies (REMARK). Br J Cancer.

