# Supplementary material for: MARCKS protein overexpression in inflammatory breast cancer
Source: Oncotarget. 2016 Dec 21;8(4):6246–57. doi: 10.18632/oncotarget.14057 (PMC5351628; doi:10.18632/oncotarget.14057)
Supplement: Supplementary file 1 [file oncotarget-08-6246-s001.pdf]

MARCKS protein overexpression in inflammatory breast cancer

Supplementary Materials

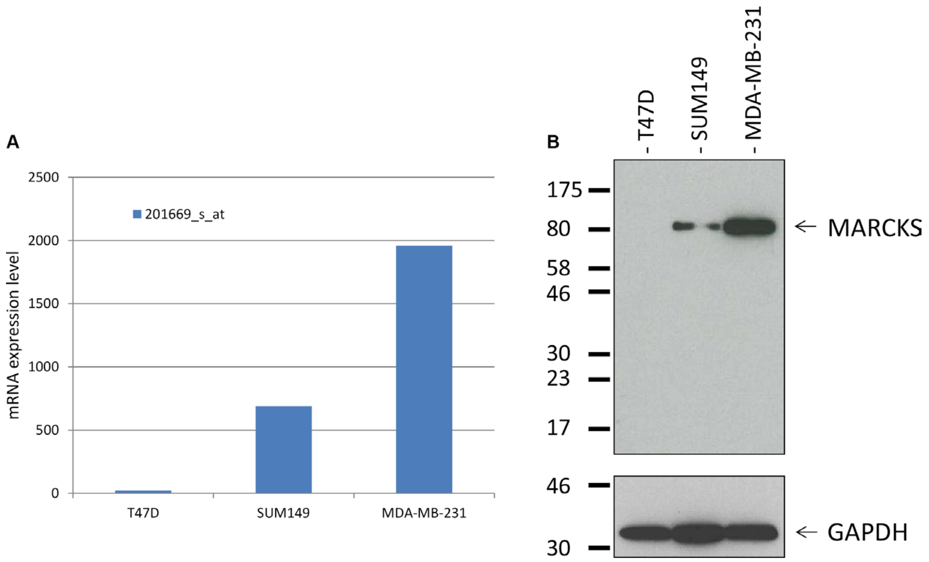

Supplementary Figure S1: MARCKS mRNA and protein expression in breast cancer cell lines. (**A**) mRNA expression level measured using Affymetrix DNA microarrays in three breast cancer cell lines with different expression levels. (**B**) Western blot analysis using the anti-MARCKS (D88D11) XP® Rabbit monoclonal Ab #5607 from Cell Signaling Technology in the same cell lines (total lysate). The antibody recognizes MARCKS (only one band at the right size) and a good correlation is observed between MARCKS mRNA and protein expression levels.

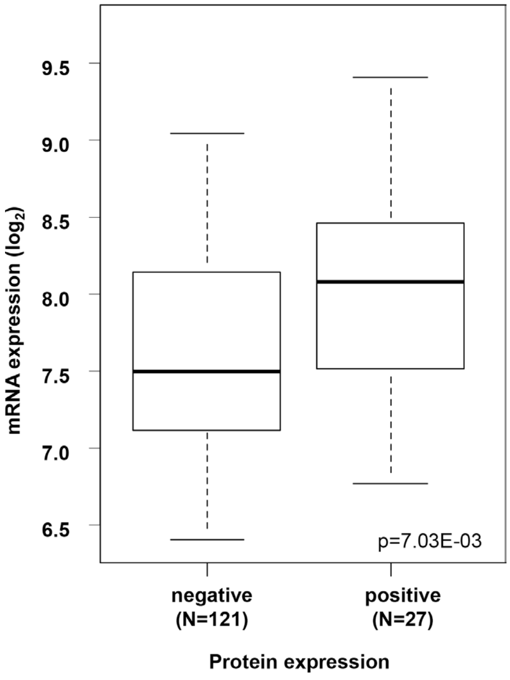

Supplementary Figure S2: Correlation between MARCKS mRNA and protein expression in breast cancer samples. Box-plots of *MARCKS* mRNA expression level according to MARCKS IHC status were established for 148 out of 502 samples (34 IBC and 114 non-IBC) that were previously analyzed by using Affymetrix DNA microarrays [7] and IHC. A good correlation is observed between MARCKS mRNA and protein expression (*t*-test).

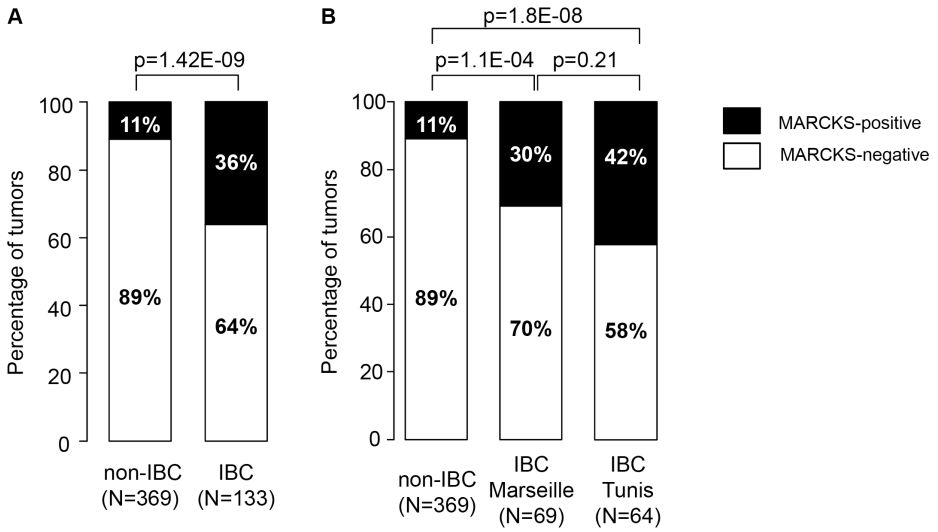

Supplementary Figure S3: MARCKS protein status according to IBC and non-IBC statutes. (**A**) Box plots showing the percentage of *MARCKS*-positive and MARCKS-negative tumors in non-IBC and IBC. The *p*-value is for the Fischer's exact test. (**B**) Similar to A, but by distinguishing the origin site of IBC samples recruitment (Marseille and Tunis).

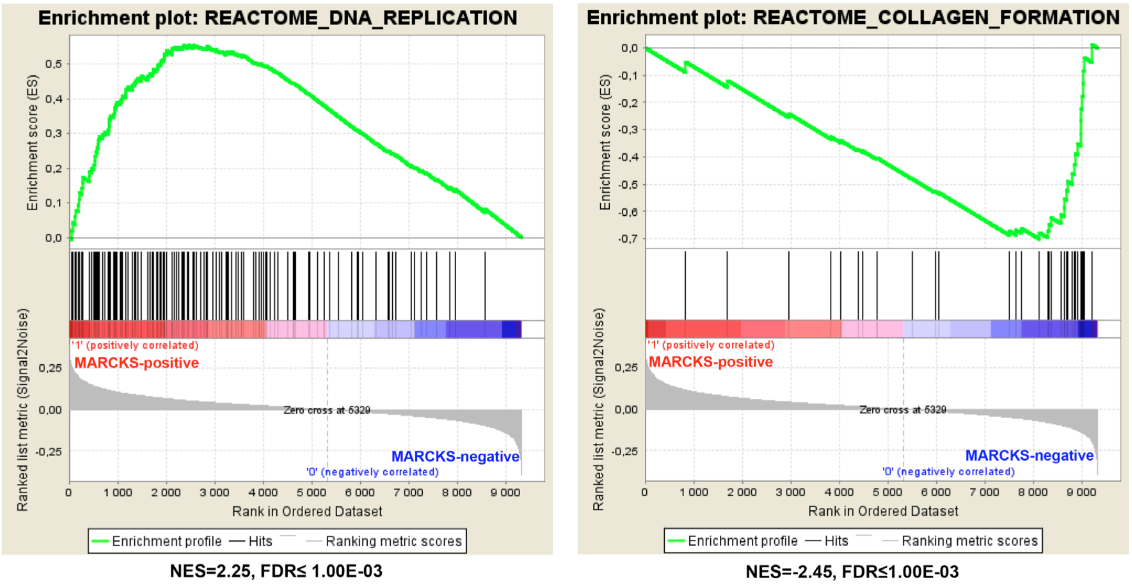

Supplementary Figure S4: GSEA analysis between gene expression profiles of MARCKS-positive and MARCKS-negative breast cancer samples. Enrichment plots for two GSEA significant Reactome gene sets between gene expression profiles of MARCKS-positive and MARCKS-negative breast cancer samples.
